# Organizational closure through regenerative signaling as a possible basis for conscious awareness

**DOI:** 10.3389/fncom.2026.1806695

**Published:** 2026-07-24

**Authors:** Peter Cariani, Janet M. Baker

**Affiliations:** 1 Harvard Medical School, Boston, MA, United States; 2 MIT Media Lab, Cambridge, MA, United States

**Keywords:** anesthesia, autopoiesis, consciousness, global neural workspace theory, neural coding, recurrent processing, temporal coding

## Abstract

Along the lines of neuronal global workspace theories, the paper hypothesizes that active neural signal regeneration in recurrent, re-entrant circuits could constitute an organizational basis for states of conscious awareness. In this view, brains are self-production systems that regenerate their own informational, signal states (“neural autopoiesis”). States of awareness themselves depend critically on sustained regeneration of sets of neural signals in local and global circuits. The set of regenerated signals at each moment determines the specific contents of consciousness. Two hypotheses are proposed. H1: Organizational closure and with it, awareness, is achieved when regenerative, self-production loops are completed, such that a stable set of signal productions is sustained. The organization of informational processes stabilizes and closes on itself. H2: Neural coding is critical for signal regeneration. Neural signals must be properly encoded in order for them to be regenerated and thereby affect the contents of awareness. In addition to simple signal suppressions at their points of origin, one means by which general anesthetic agents may abolish awareness is by scrambling neural signals. Rendering internal control signals incoherent by altering within- and across-neuron rate profiles and/or temporal patternings may disrupt signal regeneration in local and global circuits. The two hypotheses are agnostic with respect to neural coding. Our own signal-centric time-domain (TD) brain theory framework is presented to illustrate how signal regeneration could be mediated by local and global temporal codes and neural temporal processing networks. Similarities and differences between TD, global neuronal workspaces (GNW), recurrent processing (RP), integrated information (IIT) and predictive processing (PP) are discussed. Empirical testing will necessitate solving the neural coding problem at multiple system levels. This will involve investigating correlations and causal linkages between regeneration of tracked neural signals and states of awareness as well as between alternative candidate neural codes and the contents of awareness. Rhythm-tagged stimuli and high temporal resolution neural recordings are proposed to enable tracking of specific neural signals throughout the brain under conditions of waking vs. sleep vs. anesthesia, with masking and unmasking signal/noise levels. Some philosophical comments regarding organization as a potential basis for consciousness are made.

## 1 Organization of topics

The main argument in this paper is that regeneration of neural signals in recurrent loops may constitute a neural, organizational prerequisite for conscious states. The contents of awareness correspond to the sets of structured, neuronal signals that are being actively regenerated in global and local loops at any given moment. Analogous to self-production network theories of living organization (autopoiesis, autocatalytic networks), this “neural autopoiesis” hypothesis invokes a self-production network of actively regenerated neuronal signals that support sustained informational and experiential states. Whereas life depends on regenerated material components and their self-sustaining organizations of cyclic processes, awareness is hypothesized to depend on regenerated neural signals implementing informational distinctions. Once organizational closure is achieved, then such systems and their informational states become self-sustaining. This may be the fundamental reason that sustained, recurrent processes are essential for brain functions and awareness, such that the regenerative process might constitute the neural requisites for states of awareness.

This paper presents two general hypotheses regarding the neural basis of conscious awareness.

*H1:* Organizational closure through active signal regeneration is necessary for conscious awareness.

*H2:* Scrambling of signals by disrupting their structure may prevent signal regeneration and with it preclude sustained states of awareness.

The paper begins with a general introduction to what is to be explained (section 2). This includes what general patterns of neural activity are necessary for supporting states of awareness and what specific patterns of activity result in specific experiential contents of the states. Concepts related to neural coding and its relation to these two major problems are then introduced (section 3).

The concept that neural signals might require specific structure for their regeneration (hypothesis H2) is discussed in section 4. If specific structuration of neural activity is required, then anesthetic agent may abolish awareness by scrambling signals. In its most general form, this hypothesis does not depend on the nature of the specific structurations, i.e., which kinds of neural codes constitute the signals of the system. The codes could involve across-neuron patterns of firing rates or temporal patterns of spikes.

The concept that states of conscious awareness might depend on active, sustained signal regeneration (hypothesis H2) in neural self-production systems (“neural autopoiesis”) is introduced in section 5. As with H1, this general principle can apply to neural signals irrespective of how they are coded.

Relations of signal regeneration (neural autopoiesis) to global neural workspace (GNW) theory are taken up in section 6. Our signal-centric, time-domain (TD) theory of brain function is outlined in section 7. It incorporates regenerations and interactions of temporally-coded spike pattern signals in local and global delay loops. We think of it mainly as a time-domain analog of both neural global workspace (GNW) and recurrent processing (RP) theories. Similarities and differences with global neural workspaces (GNW), informational complexity (IIT), recurrent processes (RP), and predictive processing (PP) theories are covered in subsections.

Methodological and experimental suggestions for how the two basic hypotheses (H1 and H2) and the time-domain (TD) theory might be empirically tested are discussed in Section 8. Some basic philosophical differences that underlie the major theories of consciousness are considered in Section 9. Finally, general conclusions are presented in section 10.

## 2 Neural basis of awareness: what is to be explained, and in what terms

Since ancient times, philosophers have pondered the material basis of conscious awareness. In recent centuries with the rise of neuroscience, the traditional, philosophical treatment of the mind-body problem has shifted to its neural basis. Conscious awareness is most clearly distinguished as the subjective difference between waking and anesthetized subjective states. Subjective states of full and partial awareness can be further distinguished by their experiential contents (different percepts, thoughts, emotions, memories, motivations). To our knowledge, the vast preponderance of empirical neurophysiological and psychological evidence suggests that conscious awareness and its specific contents depend on neuronal activity that is organized in specific ways. The evidence suggests that patterns of neuronal activity in the form of trains of action potentials are necessary for conscious awareness and determine its experiential contents.

Arguably, all questions of existence, including the question of why conscious awareness should exist, the so-called Hard Problem, are metaphysical questions that are not amenable to empirical, scientific investigation (see section 9, Philosophical Considerations). These methods involve observation, hypothesis creation, theory formulation, construction of predictive models, and testing through controlled experiments (Cariani, 2022). What can be investigated, are those material, neural processes and conditions as well as psychological functions and behaviors that correlate with presences and absences of our own subjective awarenesses and their specific contents at different times.

The ultimate goal of a neuroscience of consciousness is to move past correlation to elucidate those specific causal material, neural conditions that are necessary and sufficient for producing (1) states of awareness and (2) their specific contents. In other words, what are the neural requisites for being aware and having the capacity to make any distinctions vs. those that enable making specific distinctions. These two complementary sets of fundamental questions were differentiated by neuroscientists as “the general operation of consciousness” (GOC) and “content of consciousness” (COC) (Thatcher and John, 1977; Thatcher, 1997). Over the last several decades (e.g., Koch et al., 2016), they have come to be widely known, respectively, as identifying the “neural correlates of consciousness” (NCCs) and “neural correlates of the contents of consciousness” (NCCCs).

### 2.1 States of awareness

States of awareness encompass full, partial, and absence states that range from alert and attentive, full waking consciousness to those of partial awareness (e.g., meditative, hypnotic, trance, inattentive daydreaming reverie, hypnopompic and hypnogogic states, various stages of sleep, lucid dreaming, parasomnias (Warren, 2007) to those of minimal awareness (some comatose, epileptic, and minimally-conscious states, emergence from anesthesia (Mashour and Alkire, 2013) to the complete absence of awareness [deep general anesthesia (Mashour, 2024), deep coma, seizures, brain death]. Some neurological conditions such as syncope/fainting/passing out, absence seizures and some minimally conscious states produce intermittent, transient complete losses of awareness.

### 2.2 Contents of awareness

The contents of awareness encompass all of the conscious, supraliminal distinctions that can be made. In our usage of the term, these distinctions go well beyond traditional restrictive philosophical definitions of qualia to include different percepts, cognitions, emotions, memories, motivations/desires, and action choices. Perceptual distinctions are perhaps the easiest to characterize because much, if not most, of perception is driven by external stimuli such that distinctions involving detections or discriminations, such as seeing a blue triangle vs. a red circle usually have physical correlates that have public-observables that are unambiguously distinguishable and quantifiable. An example would be psychophysical measurements of Weber fractions for discriminating pure tone frequencies or sound intensities. The neural correlates of such perceptual distinctions are generally easier to identify than those related to internal cognitive (e.g., concepts, word meanings), emotional, motivational, or mnemonic states (memories) that are less clearly delineated.

## 3 Neural coding and conscious awareness

We believe that solving the neural coding problem within central stations is critical for understanding the specific contents of our experiences (NCCC’s). However, with some notable exceptions (John, 2001), most neural theories of conscious awareness have focused on accounting for finding correlates of states of consciousness (NCC’s) rather than for the specific neural patterns that are responsible for different contents of awareness (NCCC’s).

As we conceive and define it, the neural coding problem involves the understanding of what specific aspects of neural activity are responsible for the various informational functions of brains (Perkell and Bullock, 1968; Cariani, 1995; Cariani and Baker, 2025). Understanding the auditory modality by itself, only one corner of the brain, is quite a complex and daunting enterprise (Cariani and Micheyl, 2012).

To paraphrase Gregory Bateson, what are the neural differences that make (meaningful, functional) differences? What patterns of neural activity enable brains to make distinctions that can switch internal functional states and external behaviors? These can be differences in states related to sensory, perceptual, cognitive, motivational, emotional, mnemonic, deliberative, action-choice, and action alternatives. Within each of these functionalities and modalities we seek to understand the particulars of all the distinctions that they make, i.e., how each one works.

**FIGURE 1:**
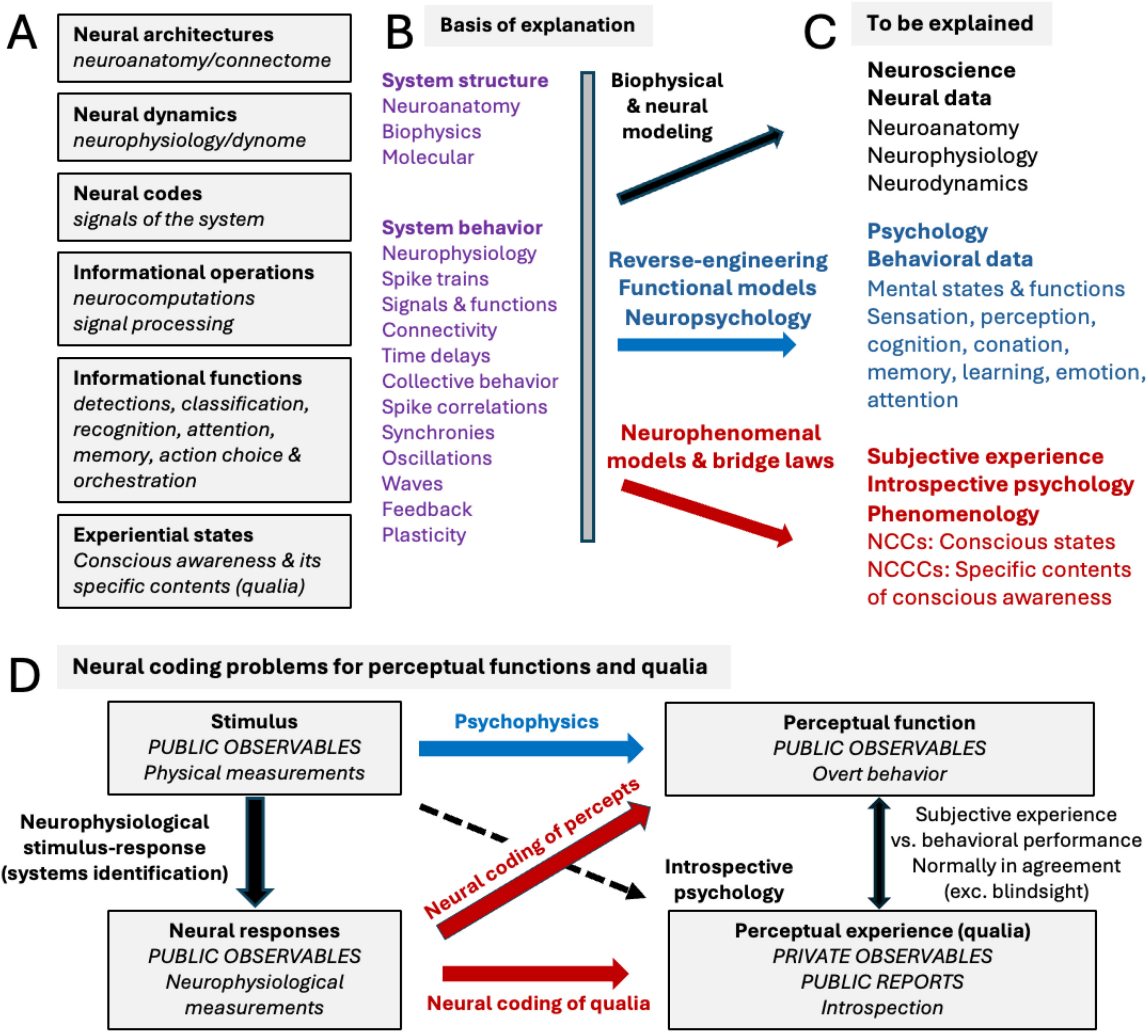
The general problem of understanding how nervous systems work as biophysical (black), informational (blue), and experience-producing (red) systems. **(A)** Different aspects of the problem. **(B,C)** What is to be explained (*explananda*, **C**) in what terms (*explanans*, **B)** neural modeling (black) predicts neural behavior in terms of the behaviors of individual neurons, ensembles, and populations. Reverse-engineering (blue) seeks to discover the functional principles that are operant in nervous systems using information processing models that account for mental functions. Neurophenomenal models and bridge laws attempt to predict subjective experiences, including different states of awareness and their phenomenal contents, from neural behavior. **(D)** The neural coding problem as it relates to the neural basis of perceptual functions and subjective experience. Top panels were updated from Cariani and Baker (2022).

As with the generation of conscious experience, the neural coding problem is an essential part of the project of formulating a complete description of what brains are and what they can do (Figure 1). The description thus needs to encompass the structure and behavior of the parts and their organization, how it achieves its informational functions (reverse-engineering), and what experiences are generated (phenomenology (Figures 1A–C).

Neural coding in the central nervous system and the neural basis of consciousness are arguably the two most fundamental open questions in neuroscience today, despite the very few scientists explicitly tasked with studying them. Many practicing neuroscientists believe that the problem has essentially been solved, consequently, there is no need to tackle it. At levels near sensory and motor surfaces, it has been largely solved for specific percepts and actions. However, as far as we know, at neural stations far removed from these surfaces (cortex, basal ganglia, limbic structures) the coding problem remains unsolved. Although huge advances have been made in understanding mechanisms of attention and memory, how specific attributes are encoded in memory traces (Lashley’s engrams), remains elusive. Without a firm understanding of the nature of the “signals of the system,” most neuroscience is in a position not unlike that of biology before the discovery of the genetic code in DNA. And without an adequate theory of consciousness, we don’t yet understand the most essential aspects of our subjective existence.

With respect to consciousness, neural coding consists of two different, but related sets of questions. Figure 1D shows these questions in the context of perception. First, what patterns of neural activity subserve informational functions (neural coding of percepts, upper arrow, a.k.a. “access consciousness”)? Second, what patterns produce differences in experience [neural coding of qualia, lower arrow, “phenomenal consciousness” (Block, 2005)]?

Some theories, such as GNWs, focus on explaining access consciousness and functions, whereas others, such as IIT and RP, focus on how phenomenal consciousness is produced. This dichotomy of purpose causes confusions when attempting to compare, contrast, and evaluate the various theories (Seth and Bayne, 2022; Storm et al., 2024; Mudrik et al., 2025a; Mudrik et al., 2025b).

For the most part, distinguishing access from phenomenal experience is not problematic. Under favorable, controlled psychophysical experimental conditions (fully awake/attending, unambiguous stimuli well above thresholds, elimination of context-related primings, qualia-matching tasks) what we are aware of (and can respond to or report) and what our brains can discriminate can be rigorously quantified. These methods also avoid issues related to experiencing vs. reporting (cf. Lamme’s comments on reports (Mudrik et al., 2025a).

## 4 Neural coding and anesthesia

A working assumption is that for neural signals to be successfully propagated and regenerated within the system their constituent patterns of neural activity must be organized in a form that is compatible with the system such that the patterns play identifiable informational roles. Not every spike is necessarily meaningful, i.e., a “difference that makes a difference,” in either experience or function.

If awareness depends on neural activity that is organized in a particular way, then there are several means by which the coherence of the activity, and hence its organization, could be disrupted, and with it, awareness would be abolished. These failure modes include (1) signal suppression, (2) disruption of coherence of signal processing, including binding processes, and (3) scrambling of the signals themselves. Note that these different modes are not mutually exclusive, and combinations of these processes have been proposed (John and Prichep, 2005). Multiple cellular sites of actions might also be possible: some classes of anesthetic agents might disrupt neuronal functions at synapses, whereas others might act on axons. Most of the current literature focuses on synaptic effects (Hao et al., 2020) and molecular, e.g., lipid membranes vs. their embedded proteins (Hansen, 2025). Despite different cellular sites of action, there could nevertheless be commonalities on molecular levels in how anesthetics affect ion channels in synapses and axons.

First, general anesthetic agents might suppress the signals of the system, however coded. In accord with channel-coding assumptions, most discussion of the functional effects of anesthetic agents in the current literature assume that alterations of synaptic functions suppress neural firing rates in critical stations and/or alter the relative activation levels of different neural populations. They focus on molecular targets of anesthetic action and on changes in synaptic function that would presumably alter neural firing rates. However, there are many reasons to question whether rate suppression is the only means by which anesthesia might abolish conscious awareness There are known general anesthetic agents, such as chloralose, that increase neural firing rates in what are thought to be critical neural populations. For a comprehensive review of theories and mechanisms of anesthesia as they relate to neural network functioning, and critique of early, simple “wet blanket” signal suppression theories, see (Mashour, 2024).

Second, anesthetic agents could disrupt the coordination of signal processing. Current “communication through coherence” theories posit attentional and working memory mechanisms whereby processing of neural signals can be controlled by means of oscillatory synchronies and phase couplings. Anesthetic agents have been observed to disrupt phase alignments of population-wide oscillations between interacting cortical populations (Bardon et al., 2025), thereby also impairing these coherences. Similar kinds of disruptions of neural dynamics appear to occur with different anesthetic agents (Eisen et al., 2026).

Third, general anesthetics might abolish awareness by scrambling the basic signals of the system. To the extent that specific neural signals are based on characteristic firing rate profiles, then altering these profiles, even without changing average firing rates of populations, could make those signals functionally meaningless to the system. Scrambling signals rather than simply reducing firing rates might explain why changes in firing rates might not always be good predictors of levels of anesthesia and of awareness.

To the extent that temporal spike codes are involved, agents that disrupt the temporal structure of neuronal responses would similarly scramble these signals (Cariani, 2000a). To the extent that temporal codes based on spike latencies re: population oscillatory phases (Cariani and Baker, 2022; Hopfield, 1995), then disruption of neural oscillations will prevent decoding of information-bearing spike latency patterns. General anesthetics have been long known to reduce higher frequencies observed in cortical population responses, both for phase-locked stimulus-driven (Goldstein et al., 1959; Jasper, 1965) and endogenous oscillatory rhythms (Brazier, 1972; Nunez, 1995; Alkire, 1998; Eisen et al., 2026). These reductions may be indicative of general degradations of spike timing that could disrupt temporal coding both in spike trains of individual neurons and in collective population responses, as observed in gross potentials. Because gross potentials reflect only the synchronized component of population responses, if individual spike trains are jittered, then interneural synchronies will be reduced, with the effect of reducing oscillatory peak amplitudes and low-pass filtering their spectra.

It bears mention that many neurons have a superexcitable, a.k.a. supernormal, phase of post-action potential recovery in which it is easier to re-excite the neuron during a particular period after it has fired. In other words, they have a “sweet spot” for re-exciting them, i.e., a temporal resonance. Such phases are widely found: in peripheral and central myelinated and unmyelinated axons in cerebellum (Gardner-Medwin, 1972), as well as in callosal fibers of visual cortex (Waxman and Swadlow, 1976) and hippocampus (Bartesaghi, 1987). When gas anesthetics are administered at their effective concentrations for inducing general surgical anesthesia, superexcitability, as measured in threshold recovery functions of sciatic nerve neurons, is abolished (Raymond and Lettvin, 1978; Raymond, 1979; Cariani, 2000a).

If the contents of awareness are mediated by temporal codes (NCCCs) and these codes depend on processing that uses these temporal resonances for their interpretation, then the contents of neural signals (their meanings to the system) will be scrambled (mode 3). On a system level, abolition of superexcitable phases could also disrupt the coherence of coordinations between different neural populations (mode 2). All of these might inhibit regeneration of neural signals in global circuits, reducing firing rates (mode 1) and precluding the sustained, coherent neural informational states that enable states of awareness (NCCs).

## 5 Neural autopoiesis

**FIGURE 2:**
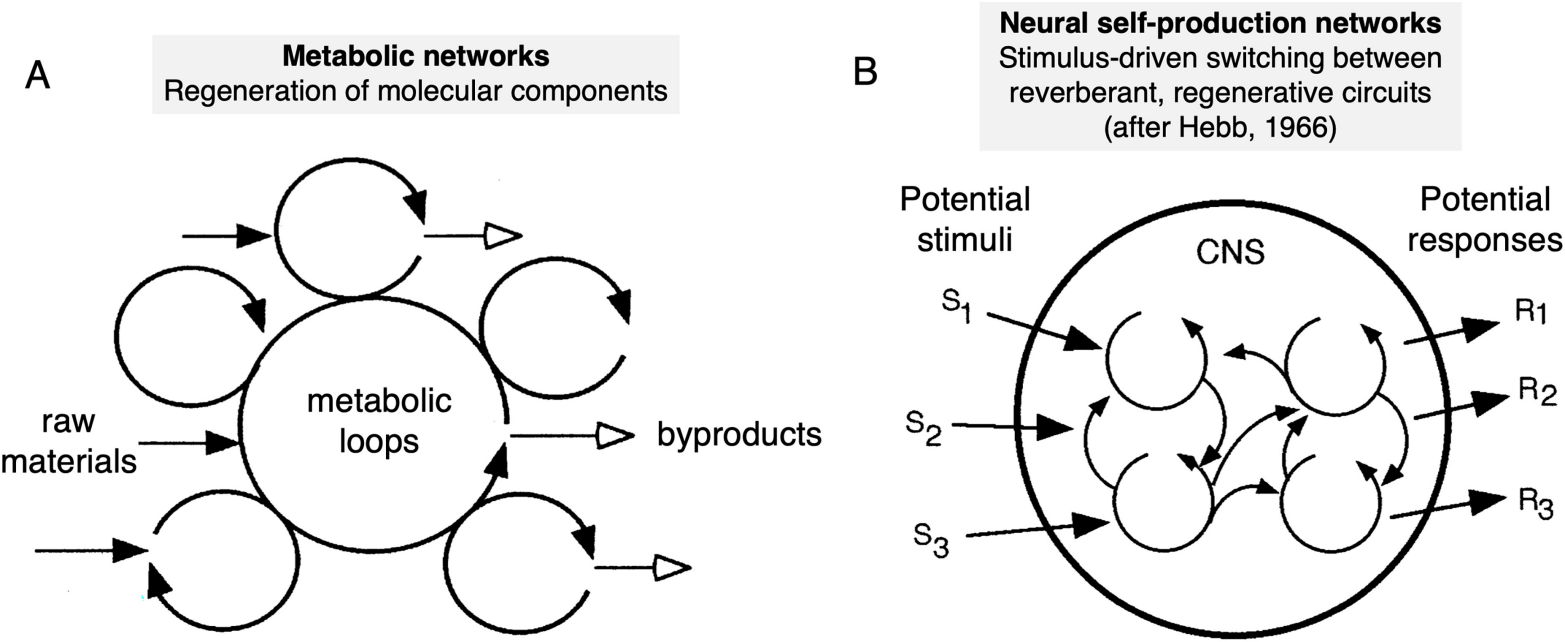
Metabolic and neural self-production systems, after (Cariani, 2001). **(A)** Metabolic systems that produce their components. **(B)** Neural signal self-productions in reverberating loops, after (Hebb, 1966).

The concept of self-producing systems (Mingers, 1995) may be of use to neural global workspace theories in explaining why recurrent pathways and signal regeneration might be crucial. Self-producing systems, autopoietic systems (Maturana and Varela, 1973; Maturana and Varela, 1980; Maturana, 1981; Mingers, 1991; Varela, 2025), hypercycles and autocatalytic networks are related ideas that have come from cybernetics, general systems theory, and theoretical biology. They have counterparts in theories of neural networks (Figure 2).

What is novel about self-production (autopoietic) systems beyond conceptions of simple, isolated production cycles is that they consist of networks of sustained mutually-supporting productions that result in organizational stability for the whole network. Neural autopoiesis is novel in that it highlights networks of mutually-reinforcing neural signal productions.

We hypothesize that conscious awareness may be a concomitant of neural signal self-production systems. Such systems are capable of sustaining provisionally stable informational states, i.e., states that can make distinctions that switch internal mental states and external behaviors. The neural signals consist of spike trains. Those neural signals that are regenerated and stabilized by global loops constitute the neural concomitants of the contents of awareness.

This idea can be described as a “neural autopoiesis” (Cariani, 2000b), in analogy with the concept of autopoiesis from theoretical biology. Autopoiesis is a theory of the essential relations that define what is a living organization. The process entails ongoing self-production of material components and relations. Networks of production processes regenerate material parts, while preserving their organization. In neural autopoiesis, sets of mutually-reinforcing neural signals rather than cellular components are actively regenerated (Cariani, 2000c,^2001^).

In cellular autopoiesis physical boundaries, cell walls, are critical constraints that allow each cell to retain its own components. In neural autopoiesis the boundaries involve which neural signals are actively regenerated within a local and/or global network. In neural systems these boundaries change with changing sets of regenerated signals. If only certain types of neural structures (e.g., thalamic, cortical, striatal, hippocampal neural populations and circuits) are able to regenerate signals, then the structural boundaries of a neural self-production system would be demarcated by these neural populations. This is similar to the IIT theory in that only some networks have internal connectives that support sustained neural activity and integrations (e.g., not the cerebellum). RP and TD theories would agree that only some populations and networks can support signal regeneration and integration (via recurrent circuits), but it may be fair to say that the working assumption of both theories, and perhaps also some varieties of GNW as well, is that these capabilities are not restricted to particular cortical regions.

Organizational closure involves the closing of production loops within a network of processes. Organizational closure and stability is achieved when essential production processes form closed loops. In the neural case, sustained regenerations of neural signals and their experiential concomitants require recurrent pathways and their transmission through at least one full cycle (closure). Once a full cycle is achieved, the process resets its initial operating state, such that the cycle can be repeated and self-sustained. In the evolution of factory assembly a century ago, the assembly line transformed linear chains of productions into networks of cyclic, repetitive processes (Giedion, 1948).

In the GNW theory, the initiation of this completion is called “ignition.” However, in all GNW theories, including ours, it is not the ignition event itself but the ensuing self-sustaining regeneration of circulating signals that would be the necessary and sufficient requisite for states of awareness. To use a motor analogy, starting an engine is necessary for initiating its continued running, but it is the continued cyclic motion that enables the engine to move the vehicle.

The organizational closure of autopoiesis has its antecedents in theoretical biology and cybernetics. Closing feedback and production loops is central to classical cybernetics, with the reverberating circuits of Lorente de Nó and Fulton (1933/1949) and the “nets with circles” of McCulloch and Pitts (1943) and (Hebb, 1949), the “inverse feedback loops” of McCulloch (1951) and reverberating activity in re-entrant loops (Edelman, 1992; Llinas et al., 1998; John and Prichep, 2005; Edelman and Gally, 2013). In the context of living systems, Maturana and Varela linked organizational closure with structural stability and autonomy (Maturana and Varela, 1973; Maturana and Varela, 1980; Maturana, 1981; Varela, 2025). Informational closure in the form of a system that actively chooses which inputs on which to act, has also been invoked (Llinas and Pare, 1996). Despite their structural closure by virtue of their self-productions of cellular components, nervous systems nevertheless are informationally-open to their environments. While they control which inputs, i.e., which sensory channels, they pay attention to and act upon, they do not control the specific readings that come through them. A scientist chooses which sets of measuring instruments to use, but does not determine what the specific measurement outcomes will be.

These ideas also animated more abstract, cybernetic conceptions of consciousness based on organizational closure through iterated reciprocal, and evolving communications, a.k.a. “conversations” (Pask, 1979, 1981). Self-organized stable patterns of communications, interpretations, and behavioral interactions (“eigenstates”) between autonomous, self-constructing percept-coordination-action agents form the basis for so-called “second-order cybernetics” (Foerster, 2003).

In dynamical systems terms, the components themselves are stable attractors, and the conditions for their sustained production can be modeled in terms of stable (attractor) limit cycles of production states. Dynamical systems can shed precious light on the mechanics of microprocesses that produce more macroscopic behavior patterns (Kopell et al., 2014), but if the models become too complex, they can lose interpretability and meaning. Focus on high dimensional trajectories through neural phase spaces can also obscure functional, engineering principles that need to be grasped in order to reverse-engineer biological neural systems. In our view, mixtures of low level neural dynamics, high level psychological functions, and mid-level neural signal processing models will all be needed for understanding how brains work.

Pervasive correspondences between metabolic networks and neural networks have long been appreciated by theoretical biologists and theoretical neuroscientists (Katchalsky et al., 1974). Organizational stability in both types of networks depends on cyclical production processes (Figure 2). Life consists of cycles, and brains are nothing if not networks of cyclical, recurrent, re-entrant signal transmission paths. In both metabolism and biological neural networks there are simple self-production cycles and more complex cycles consisting of control of simple cycles. In the cell there are enzymes that switch on or off particular metabolic cycles and control cycles involving gene expression. Likewise, there are neural cycles consisting of re-entrant loops, such as thalamo-cortical, cortico-cortical, and limbic circuits, that can be switched by prefrontal- and basal ganglia-mediated control circuits, depending on the current system-goal, the task-at-hand.

A notable difference between metabolic and neural cycles is that metabolic cycles consist of repeating sequences of transformations of molecular components, whereas cycles of neural signal productions regenerate their component neural signals in parallel. With exceptions such as prions, in metabolic networks, the components are not self-catalyzing, whereas for neural signals they can reinforce and replicate themselves. Each neural signal can replicate itself via a recurrent signal path.

One can regard and use autopoiesis either as a heuristic metaphor, as a set of design principles, or as a means of recognizing systems consisting of self-production cycles. Autopoiesis enables design of self-repairing systems that have organizational integrity, structural stability, and functional resilience. Recognizing a self-production system in nature involves defining what are the components, what are their production relations, and whether cycles of production, either sequential or parallel, can be identified in its state-transition structure.

It might also be thought that the idea of neural autopoiesis might not add anything to brain science that is not already implicit in dynamical systems or physics, but, analogously, do we think that the idea of the reaction cycle and networks of mutually supporting cycles adds nothing to biology that is not already implicit in chemistry?

Some might think that the concepts of neural self-production and organizational closure through regenerative signaling proposed here are implicit in the previous ideas mentioned above and are therefore not entirely new. We concur, but believe that the use of this terminology that ties them together is new and conceptually useful. In any case, these phrases serve to alert us to and provide yet another perspective from which stable system- and network-properties can be contemplated.

## 6 Global neuronal workspaces (GNWs)

In recent years there has been increased comparison between different theories of consciousness (Lamme, 2018; Seth and Bayne, 2022; Mudrik et al., 2025a). Of the neurally-grounded ones on the current scene, global neuronal workspace (GNW) theories and recurrent processing (RP), theories appear to us to be the strongest candidates. Although we in agreement with many of its philosophical and methodological positions (see sections 6.1.3 and 8), we are less certain of the neural grounding assumptions of Integrated Information Theory (IIT). This is perhaps because of its abstract nature and its commitments to particular neuronal loci as seats of awareness.

Although modern neuroscience has been grappling with questions of neural integration, neural coding, and their relations to the structure of conscious experience for a century or more (Troland, 1929; Boring, 1933, 1942; Gerard, 1959), major inspiration for global neuronal workspace theories came from the integrative cognitive workspace theory of Baars (1988).

Neural workspace and recurrent processing theories of consciousness are based on coherently-organized, distributed neural activity patterns, as opposed to anatomical localization theories (Rose, 2006) that depend on activation of particular neural populations (Popper and Eccles, 1977; Koch and Crick, 1994; Koch, 2004; Koch et al., 2016). This division parallels older, but ongoing debates between localizationist and distributed theories of informational representations in brains (Lashley, 1960; John, 1972; Orbach, 1982; Orbach et al., 1998).

Theories based on distributed organizations of neural activity include neural field theories, temporal correlations and population statistics of spike timings (John, 2001, 2005), neural global neural workspaces (Dehaene, 2001, 2014; Dehaene et al., 2014; Mashour et al., 2020), activations of recurrent pathways (Lamme and Roelfsema, 2000; Lamme, 2006), cortico-cortical re-entrant loops (Edelman and Gally, 2013) and parietally-anchored re-entrant loops (Pollen, 1999; Pollen, 2003, 2008, 2011). Surveys of these and alternative theories can be found in the literature (Rose, 2006; McGovern and Baars, 2007; Dykstra et al., 2017; Seth and Bayne, 2022; Dykstra et al., 2025). See Mashour et al. (2020) for a comprehensive review of GNW that surveys its neural, psychological, and phenomenal evidential basis.

Most GNW theories incorporate some concept of “ignition,” the initiation of active, self-sustaining, self-reinforcing neural activity in reverberant circuits. Our conception of ignition entails active regeneration of neural signals in which at least some specific neural spike patterns that encode attributes of events are preserved throughout the re-entrant loop. We see signal regeneration as an ongoing process, rather than a series of discrete events. Although we focus here on network-level, macroscale-level theories for awareness, the neural requisites for network processes, such as ignition, could depend critically on particular micro-scale “circuit level” processes, e.g., activations of layer 5 pyramidal cells (Dykstra et al., 2025).

Recurrency provides a general framework for informational integration in brains, i.e., how disparate types of local specialized and detailed information can be integrated, stabilized, amplified, regenerated, and broadcast by global circuits. GNW, RP, and TD theories explicitly hold that recurrent, re-entrant pathways are needed for sustained neural informational states. Early versions of IIT also explicitly included re-entrant connections along with the informational complexity measure as core ideas (Storm et al., 2024): “a group of neurons can contribute to conscious experience only if it is part of a functional cluster that achieves high integration through reentrant inter-actions in hundreds of milliseconds.” In later versions of IIT, these evolved into “cause-effect” structures consisting of (dense, re-entrant) “grids” (Seth and Bayne, 2022).

Why is recurrency thought to be critical? Two reasons are given by Mashour et al. (2020) concerning

“why conscious processing might rely on recurrent loops between distributed processors. First, recurrent connections can help amplify a signal through recurrent excitation, thereby making it available for other cortical processors. Second, recurrent loops can sustain a signal, e.g., such that it could be maintained in working memory” (p. 782).

To add to this, recurrency facilitates the amplification of neural signals in local circuits, stabilizes them, and allows them to propagate into global circuits. In order to self-propagate and regenerate in local and/or global circuits, they need to exceed some threshold. In effect, recurrency can amplify local signals such that they can overcome inhibition and break through to enter global circuit workspaces. If the neural signals entering global circuits retain some temporal structure (see rhythm-tagged stimuli in section 7), then the recurrent signal may act as a matched filter that selectively amplifies incoming neural signals with similar temporal structure.

Blindsight is a pathological situation in which informational access is preserved in the absence of accompanying “phenomenal” awareness (Block, 2005). GNW theories would explain the consequent lack of awareness of visual features in terms of failures to “ignite” global circuits that then could sustain the neural patterns that would encode them (Silvanto, 2015). Early on Pollen proposed that recurrent excitations within the cortical visual pathway chain might be necessary for visual awareness (Pollen, 1999). Lamme refined this hypothesis (Lamme, 2001; Lamme, 2006) to include occipital-parietal-occipital re-entries. Pollen’s later, “anchored-loop” theories would point to the necessity for recursion, but also a necessity to locate visual features in relation to parietal representations of self (Pollen, 2011).

## 7 Time-domain theory of brain function (TD)

We have proposed our own, tentative time-domain (TD) theory of brain functions based on signal-signal interactions and active regenerations of temporally-coded neural signals (Cariani and Baker, 2022; Baker and Cariani, 2025; Cariani and Baker, 2026). Our previous work on temporal coding of pitch, consonance, and timbre in the auditory system (Cariani and Delgutte, 1996; Cariani, 1999; Tramo et al., 2001; Cariani, 2019) and time-domain analyses of speech (Baker, 1975) has led us to survey the extent of such codes in other sensory modalities (Cariani, 1995; Cariani and Baker, 2025) and to seriously consider the possibility of multiplexed time codes in the central nervous system.

For several decades we have been following the evolution of brain theories of consciousness, and our thinking has co-evolved with this field. As a result, organization of high-level processing in the time-domain (TD) theory has many commonalities with global neural workspace (GNW) and recurrent processing (RP) theories. We consider the TD theory as it relates to conscious awareness to be a time-domain version of a mixture of these theories. As in those theories, we hypothesize that active neural signal regeneration in recurrent, re-entrant circuits is the organizational basis for both short-term working memory and conscious awareness. We assume that neural signals are multiplexed and widely broadcast in global and local circuits throughout the brain, such that, depending on current contexts and attentional mechanisms, many diverse neural populations can potentially become part of the regenerative network or be cut off from it.

**FIGURE 3:**
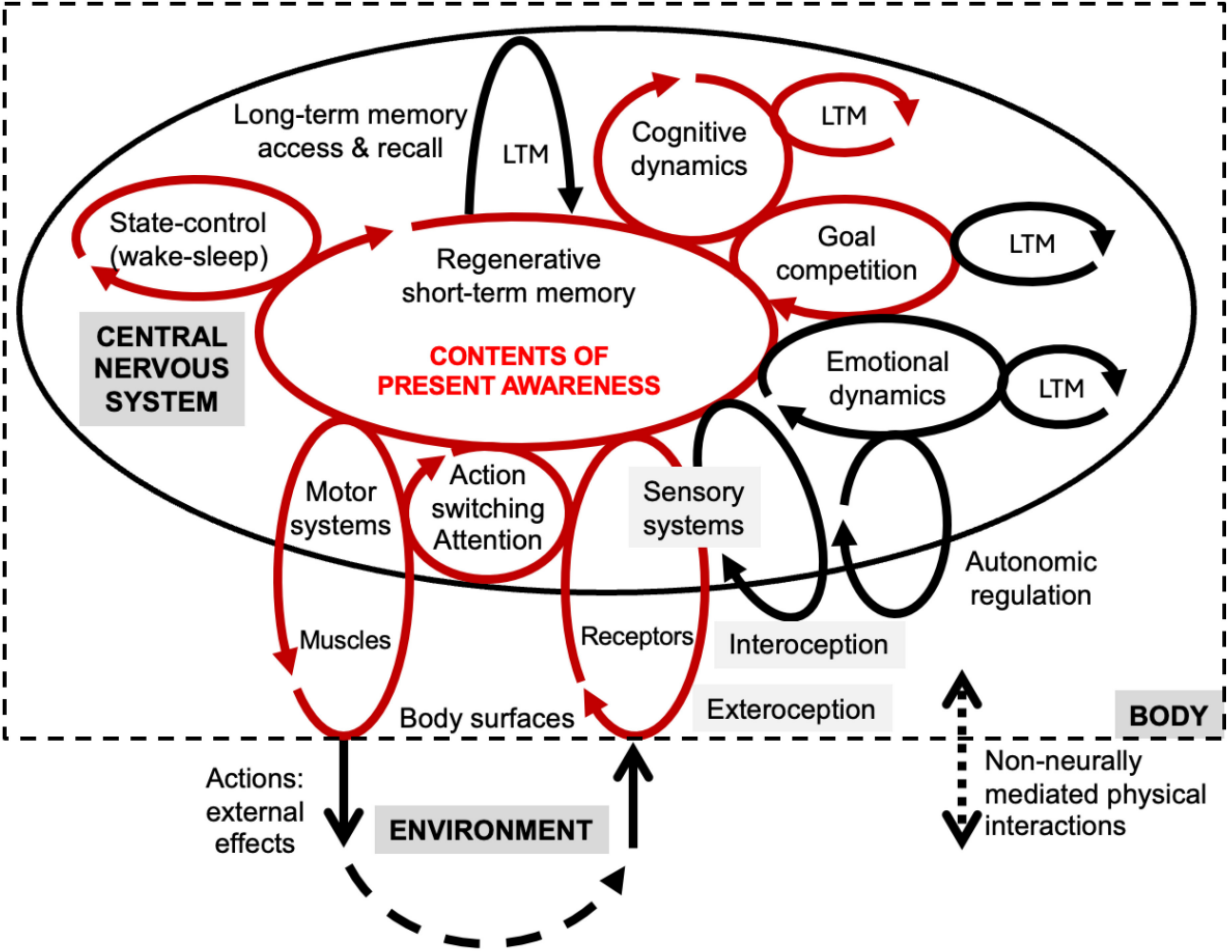
Schematic of major functionalities of nervous systems as they relate to conscious awareness. Mind-brain as a network of circular-causal informational processes in interaction with the rest of the body and the external environment. Red loops indicate those sets of functionalities and their underlying neural circuits (not shown) that would dominate signal dynamics in a particular context and whose contents would affect awareness during an emotionally un-arousing cognitive working memory task. The contents of awareness at each moment would consist of those sets of neural signals in global and local loops that were being regenerated by the whole network.

Figure 3 depicts major functionalities of brains as recurrent processes (cf. illustrations of global attentional workspaces (McGovern and Baars, 2007; Baars and Gage, 2010, Figure 8.1), reverberating networks (John and Prichep, 2005) and of global neural workspaces (Dehaene, 2001, 2014; Mudrik et al., 2025a). Each functionality loop is assumed to be subserved by corresponding re-entrant cortico-thalamic, cortico-cortical circuits, as well as those involving basal ganglia and other subcortical structures responsible for attentional, emotional, and reward functions. Cerebellar timing and coordination processes are omitted, as the cerebellum is widely regarded as a feed-forward-only system whose stimulation does not appear to directly affect the contents of awareness (Koch et al., 2016). The cerebellum therefore is thought to lie outside the set of brain structures and processes responsible for conscious states.

The TD theory envisions brains as networks of delay loops in which temporally-structured neural signals circulate and either reinforce or interfere with other neural signals. The temporal patterns that constitute the neural signals are presumably processed in neural timing networks and time-delay networks. In the theory, binding of related events and multimodal collections of object attributes could be accomplished in these networks through common timing (e.g., synchrony, common onsets of events), common temporal patterning (e.g., common rhythmic structure of events), and common spatiotemporal patternings (e.g., common oscillation and traveling wave interference patterns).

### 7.1 Comparisons with other theories

Major theories of conscious awareness were recently presented by their proponents and their theoretical commitments compared (Mudrik et al., 2025a). The set of theories included global neural workspace (GNW), recurrent processing (RPT), higher-order representations (HOT), predictive processing (PP) and integrated information (IIT). Unless otherwise noted, the comparisons below refer to the various theoretical tenets expressed in that paper.

### 7.2 Neural coding vis-a-vis theories of consciousness

Although many of these theories make tacit assumptions about neural coding, such as observed increases in firing or decreases in neural firing rates, these observables are almost invariably used only as simple indicators of neural activity and not as neural correlates of the specific contents of awareness. Very few make explicit their neural coding assumptions regarding specific contents, such as low-level perceptual attributes (as opposed to using a neural network to distinguish between presence of some percept in awareness vs. its absence). Instead, most often they tacitly rely on rate-coding assumptions that are widely held within the neuroscience community at large.

In some discussions, oscillatory neural behaviors are invoked in the context of intra- and inter-areal coordinations of information processing, and the deleterious effects of anesthetics on them (e.g., amplitude and phase disruptions, mode 2). At present, it appears that only the time domain (TD) theory posits that anesthetics might scramble the basic signals themselves that encode specific informational attributes and that enable signal regenerations.

### 7.3 Neural global workspace (NGW) theories

The time-domain (TD) theory shares with most of the major theories the basic idea of recurrent activity in global circuits. In these theories, once global loops are activated (“ignition”) they also regenerate specific sets of neural signals. There are two-way interactions between local and global circuits, as well as between neighboring local circuits that can act in either direction to reinforce or attenuate signals in the others. In cases of bottom-up activations, e.g., from salient sensory inputs, including unexpected events, strong local signals begin to drive the contents of global loops. When these signals reach some threshold in global loops, their associated phenomenal distinctions enter conscious awareness. In cases of top-down selective attention, global circuits can selectively attenuate irrelevant neural populations via basal ganglia mediated inhibitions and disinhibitions, effectively by altering local loop gains. When loop gains are negative, the local neural signals rapidly die out, whereas when they are positive, they build up. Under normal circumstances of inattention and relaxation in which no goal or task is being pursued, the local loops have a slightly negative loop gain, and signals rapidly die out. When global loops are activated, then task-relevant local thalamo-cortical loops are disinhibited by basal ganglia pathways and these task-relevant loops then have a slightly positive loop gain. If global signals have commonalities with local ones, however encoded, then global signals in re-entrant pathways can selectively reinforce local signals by means of cross-correlation operations.

In Dehaene’s global workspaces only neural signals in global circuits affect conscious awareness. This creates an informational bottleneck in which conscious visual awareness is assumed to be a highly reduced version of visual scenes that are represented in local circuits. In contrast, in TD theory, in waking states when global circuits are engaged, local and global circuits are thought to form a unified, coupled network in which the contents of both local and global circuits are being regenerated. Even if local unimodal circuits hold all the lower-level details and global supramodal ones hold coarser representations and different, higher-level attributes, the signals encoding lower-level details can enter global circuits as needed, and consequently, in TD theory as it stands, there are presumed to be no bottlenecks as such.

### 7.4 Recurrent processing (RP) theories

The time domain (TD) theory is closer to Lamme’s recurrent processing (RP) theory (Lamme, 2006; Fahrenfort et al., 2008; Lamme, 2018, 2020), which allows the possibility for local self-sustained signal regenerations via strong bottom-up inputs, such as attention-grabbing unexpected events, without prior global circuit reinforcement. Because all of the local sensory signals bearing all of the encoded details are capable of affecting awareness, Lamme’s theory also does not assume or entail an informational bottleneck.

In the time domain theory, neural response patterns in local and global circuits likely have different but overlapping temporal structures. Possible multiplexed spike latency codes, e.g., containing lower- and higher-level attributes in the sequential hierarchy of speech coding, have been proposed (Cariani and Baker, 2022). Here precise details of stimulus attributes would mainly reside in local unimodal and association cortices, whereas coarser temporal patterns associated with event onsets and offsets, attribute bindings in both local and global circuits would be related to their counterparts by common rhythms, oscillations, and/or synchronies.

### 7.5 Integrated information theory (IIT)

Tononi and Koch’s Integrated Information Theory (IIT) (Tononi et al., 2016; Tononi et al., 2025) has been a major, formidable theory in the field for several decades now. The organizational closure hypothesis and the TD theory hold that the contents of awareness are determined by signal regenerations, whereas IIT posits instead that attainment of some threshold of informational complexity or capacity is required. Although we think it possible, if not always feasible, to describe and quantify their contents (φ*^max^*), we believe that states of awareness are binary and not graded (states of arousal are a different matter). Complexity strikes us as not being the right kind of basis for awareness. In analogy to living organization, whether an organism is dead or alive depends on its ability to regenerate its parts and their organization rather than on its complexity, e.g., numbers of different types of parts. Ability to make and hold one distinction is enough to have awareness.

As we understand it (Tononi et al., 2025), IIT in its present form also is localizationist in that it postulates the necessity of activating specific neural populations, whereas the regenerative hypothesis, TD, GNW, and RP theories depend on distributed regenerated patterns of neural activity, irrespective of where they occur. However, these two seemingly mutually-exclusive ideas may be reconciled if only certain neural populations by virtue of their network and/or local interconnections are capable of regenerating signals. IIT stipulates the existence of anatomical consciousness centers to be identified in brains. Even if such centers were to be identified, we might have the right neurons, but we would still need to solve the neurophenomenological coding problem: what aspects of the activity of these neurons is responsible for producing awareness and its specific contents?

Despite this fundamental difference, we do agree with Tononi’s approach on many methodological, and philosophical points.

First, we agree that conscious awareness is an inherent fundamental aspect of the universe that each of us can observe through introspection (Tononi and Koch, 2015). Awareness and its experiential contents are produced by the actions of appropriately organized networks of neurons. Awareness is unitary. We are far from being human exceptionalists and believe that basic awareness, albeit with different Umwelts and different sets of experiential contents, is likely to be widespread throughout the animal kingdom.

Second, the goal of a theory of consciousness is neurophenomenology, i.e., to predict the structure of awareness from patterns of neural activity. “Consciousness is a structure, not a function,” and “all quality is structure,” (Tononi and Koch, 2015). We also hold that awareness has no function per se. Although the structure of our experience is mediated by neural processes that evolved to perform functions that promote survival and reproduction, so the two are very often correlated, the conscious experience itself has no function. Our job as neuroscientists studying consciousness is to identify the causal neural conditions and processes that produce particular experiences.

Regarding structure, we would say that awareness *has* dimensional structure (Boring, 1942). Its nested dimensions include sensory, cognitive, motivational and emotional modalities, attributes within each modality, and different distinguishable states of attributes. For example, the auditory modality has basic sets of attributes of objects and events (including pitch, various timbral dimensions, loudness, location, timing, duration) and for each attribute, there are different values (distinguishable qualities, distinctions). See Cariani and Micheyl (2012) for an outline of auditory percept space. Organizations of attributes (groupings, separations) are also part of the structure. The primitive distinctions constitute the atoms of experience. Red has the quality of being red because of its relational position vis-a-vis other dimensions and distinctions.

Third, we also agree that the dimensionality and number of distinctions that can be made by a neural system can, in principle, be quantified. Tononi’s integrated information measure would be a useful concept even if it could not be reliably measured. But it can be measured under suitably restricted circumstances (Tononi et al., 2025) and/or in “weak IIT” formulations (Mediano et al., 2022). Certainly, the number of perceptual distinctions for a given attribute in a specific modality (e.g., pitch, loudness, color, shape, pain) can be quantified in a given human individual under different states of awareness. Perhaps in the foreseeable future even all of the distinctions that a simpler organism is capable of making will be exhaustively described and quantified.

The signal regeneration hypothesis is agnostic with respect to the relation of awareness to informational functions. However, like IIT, the time-domain theory is not a *functionalist* theory of consciousness because awareness is not *caused* by realizing informational functions (Mudrik et al., 2025a, Table 1). The signal regeneration hypothesis, time-domain theory, and IIT are also not *computationalist functionalisms.* None of these depend on realizing “computations” or functions. Whether we should regard neural activities as “computations” depends whether we mean some logical equivalence to Hilbertian formal procedures and deterministic finite state automata (Minsky, 1967) or whether we mean anything related to signals and information processing (Churchland and Sejnowski, 1992). To most neuroscientists today, “computational neuroscience” evokes the latter, looser, sense of the word.

### 7.6 Predictive processing (PP) and higher-order (HOT) theories

Predictive processing (PP) theories likewise seek to understand how brains can operate as anticipatory systems that guide action by minimizing predictive errors. They are fine theories for this purpose. However, according to proponent Anil Seth, “…they are, in general, not (yet) “theories of consciousness” in the sense of proposing necessary and sufficient conditions for conscious experience (or specific conscious contents) to occur” (Mudrik et al., 2025a). A potential problem for PP theories of consciousness and for radical enactivist theories in general is that not all brain functions and conscious states are necessarily involved with predicting the next state of the external world or extrapolating the consequences of actions. Yes, a great deal of our conscious experiences are related to external action, but many of our internal states and associated subjective experiences, especially when no immediate task confronts us, do not involve active anticipatory preparations for action. In their universalist forms, PP and enactivist theories risk becoming motor theories of perception and consciousness. Awareness does not require action.

Higher-order (HOT) theories of consciousness define “consciousness” in terms of metacognition, self-representation (neural representations of other neural representations), and self-reference. As HOT theories are not attempting to explain basic conscious awareness, a.k.a. phenomenal consciousness, comparisons between them and more neurally-grounded theories such as GNW, RPT, IIT, and TD are difficult to draw.

## 8 Empirical testing

Throughout much of the history of the neuroscience of consciousness, theories have been developed in relative isolation, with their interactions and interchanges mostly occurring in small face-to-face scientific conferences. In recent years, however, there has been intense public debate in the literature about whether and how theories of consciousness can be empirically tested (Block et al., 2014; Gibbons et al., 2026; Melloni et al., 2023; Cogitate et al., 2025). These debates have been accompanied by systematic comparisons of the theoretical and methodological commitments, explanatory powers, and predictive successes of rival theories (Seth and Bayne, 2022; Storm et al., 2024; Mudrik et al., 2025a; Mudrik et al., 2025b; Parshall and Christiansen, 2026).

Much of it seems to be driven by assertions that some theories are inherently untestable, (unfalsifiable) such that no amount of contradictory evidence can prove them wrong. Verification is extremely important (Cariani, 2022), and some theories, such as the many-worlds interpretation of quantum mechanics, are inherently untestable and should be recognized as such. However, even theories that are provably wrong may nevertheless be heuristically useful for developing new ideas (Feyerabend, 1973).

Premature falsification, dismissal, and funding prohibitions inhibit development and consideration of new theories. In the early 20th century Wegener’s theory of continental drift was dismissed and ignored because most geologists could not conceive of any plausible mechanism that could move continents. Only after WW II, evidence of sea floor spreading accumulated, and eventually the theory was taken seriously enough to merit more observations, empirical tests, and theory-building. Like human children and adults, theories need to be treated according to their levels of maturity. In their early formative, expansive, conjecture stages they need room to explore and grow and time to refine their ideas, whereas theories in the later, evaluative, eliminative, proof stages require more definitive predictions and rigorous testing.

### 8.1 Testing neural coding hypotheses

With the maturation of several major neurally based theories of consciousness (GNW, RP, IIT) and controversy over their testability (Tononi et al., 2025), attention has shifted to how they could be empirically tested and their various predictions compared (Seth et al., 2006; Melloni et al., 2023; Cogitate et al., 2025; Gibbons et al., 2026).

Although the two hypotheses (signal scrambling, neural signal regeneration) and the time-domain theory are still being refined using available published evidence, they are certainly open to empirical test through controlled experiments in humans and animals now and in the future.

How would one go about testing them? We see the problem as a neural coding problem. The neural coding problem entails finding neural correlates of functions (neuropsychology) and phenomenal states (neurophenomenology) and then determining which aspects of neural activity causally produce these functions and states (Figure 1D). Finding the neural correlates of phenomenal awareness involves which specific aspects of neural activity, which candidate neural codes, predict experiential distinctions. This requires observation of large numbers of neural spike trains, local field potentials, and whole population responses at many brain sites.

Once strong neural correlates of simple stimuli are identified, then causality can be probed by disrupting parts of the system in various ways and assessing whether and how the interventions change the contents of experience. Various kinds of masking stimuli can be added to study their effects on putative codes and representations by comparing trials when targets are consciously perceived or not (Dykstra and Gutschalk, 2015; Dykstra et al., 2016; Wiegand et al., 2018).

Direct driving of neurons by electrical, magnetic, focused acoustic and optogenetic means allows neural activity patterns to be manipulated and their phenomenal effects assessed. In animal studies whole regions can be lesioned (Lashley, 1960) or implanted with conductive and non-conductive materials (Sperry and Miner, 1955; Sperry et al., 1955), reversibly cooled, injected with various agents, or optogenetically inactivated to study their effects on perceptual awareness. These need to be conducted with as few animals and as humanely as possible. Optogenetics have been used with success to change temporal firing patterns while maintaining the same average firing rates (Chong et al., 2020) and similar experiments have been proposed to test neural theories of consciousness (Takahashi et al., 2025).

#### 8.1.1 Solving neural coding

Ideally, the process needs to be kept as simple and straightforward as possible. We would first concentrate on identifying neural codes for basic, simple sensory/perceptual distinctions at different levels of the system, keeping the subject in an awake, alert, attending state that is as constant as possible. We would focus first on the contents of awareness, phenomenal consciousness. It may be difficult, if not impossible, to identify the neural requisites for conscious awareness (NCCs) without first having a firmer grasp of the neural correlates of some of its contents (NCCCs).

We think that coarse distinctions may well be observable in collective, population-wide responses (e.g., EEG, MEG, fMRI). We suspect that the encodings of most fine distinctions exist at the levels of individual neurons and local ensembles such that often they may not be observable in those collective responses. If so, then techniques with more temporal and spatial detail (e.g., single- and multi-unit recordings, local field potentials, ECoG, Neuropixels) may be required. In order to discover or rule out temporal codes, high temporal precisions (channel sampling rates ≥ 5 kHz) and advanced spike correlation analytical techniques will be required. Low-pass filtering and windowing of data, as is common in spectrograms, should be avoided, and instead unwindowed correlation techniques that preserve the sampling rate precisions of the analyzed signals should be used [e.g., spike timing vs. interval autocorrelograms (Chung et al., 1970; Cariani and Delgutte, 1996) and log-inverse period plots (Baker, 1975)].

All kinds of conceivable coding schemes need to be considered. Deep learning and convolutional networks may assist in finding hidden patterns of neuronal responses that highly correlate with different contents of experience. If such techniques are used, care needs to be taken to make it possible for these systems to detect patterns of spike correlations within and across neurons. If a learning network is only presented with average firing rate data, the network may not be able to detect time domain codes, such as simple interspike interval codes, more complex temporal pattern codes or oscillatory-phase-offset codes.

The neural codes for particular qualia, i.e., NCCCs, are the simplest problem: find what specific patterns of neuronal activity produce particular qualia. This is the methodology that we used to successfully predict pitch percepts of many different types of acoustic stimuli from both recorded (Cariani and Delgutte, 1996) and simulated (Cariani, 2019) spike train data of populations of auditory nerve fibers (ANF). One uses different stimuli or stimulus parameters that produce (an experience of) the same perceptual attribute (metameric stimuli). For example, sounds with different spectra, but the same periodicity (waveform repetition rate) are all experienced as having the same pitch, despite their activations of different sets of neurons in the auditory nerve. Or one could present the same sounds at different sound levels or locations, such that their pitch and timbral qualities would be unchanged, but those related to their loudnesses or apparent locations would change.

One then records neural spiking activity in response to these parametric changes while holding one perceptual attribute constant. It is then possible to identify which aspects of neuronal activity remain invariant when the experienced quale is constant and which ones change when parameters that change that attribute change.

One can then go up the pathway, “outside in, then bottom up,” to identify neural codes in various brain regions and follow the patterns of neural activity that carry information for particular stimulus attributes. It then becomes possible to track the flow of identified neural signals through central networks, in the regions that are traversed as well as the sequencing and timings of their traversals. Some of these kinds of experiments have been conducted using visual stimuli in tracking population responses associated with the brief presentation of a visual image. However, without a more detailed understanding of which neuronal responses bear specific information, it is much harder to separate out neural activity that is related to awareness from that which is not.

Once the neural coding problem is solved for peripheral and central stations, then neural signals related to particular qualia can be traced throughout the brain. One could go up sensory ascending pathways and attempt to determine neural codes, representations, and transformations at every stage of subsequent processing. This would be a difficult and lengthy task for many perceptual attributes, but there is a workaround.

#### 8.1.2 Use of rhythmic and rhythm-tagged stimuli to track NCCCs

There are stimuli, such as rhythmic patterns, whose percepts are supramodal and whose temporal patterning should be constant and observable in spike trains and population responses at all levels. Phase-locking and entrainments to simple rhythms (Nozaradan, 2014) and frequency-tagged modulated tones (Patel and Balaban, 2000) are observed widely across brain regions.

We use the word “rhythm” here in its broadest sense, to mean any coarse temporal pattern of events, repeating or not, in which different event temporal sequences can be recognized. To avoid fusion or loss of pattern coherence, this means that events should follow neither too quickly (>10 Hz) nor too slowly (<0.5 Hz). Rhythm is a form of time perception, which is supramodal. Although rhythmic pattern perception is most acute in the auditory modality, there are direct analogs in tactile perception and vision (e.g., temporal patterns of flashing lights or of object movements). Clearly all general theories of conscious awareness would benefit greatly from study of NCCCs in modalities other than vision, especially audition (Dykstra et al., 2016; Dykstra et al., 2017) and tactile perception (Grund et al., 2021). With rhythm, the same patterns can be presented via different modalities to produce phenomenal attributes that have some properties in common.

There are several advantages to using temporally-patterned simple rhythms and rhythm-tagged stimuli in probing NCCCs and NCCs. These all relate to the interpretability of neural responses associated with specific experiences (NCCCs) and to non-specific processes that affect general conditions for any conscious experience (NCCs). Neural responses specifically related to the stimuli and to their appearance in phenomenal experience can be disambiguated from non-specific responses associated with attention, access, recognitions, reporting, levels of arousal, and the like (Mudrik et al., 2025b) that confound interpretation of undifferentiated late responses such as P300 waves. The specific neural signals that are directly related to the specific perceptual experience can be tracked from one region to another. At least some issues related to informational bottlenecks and the contents of local and global circuits could be resolved.

One can also estimate the strengths of stimulus-related neural signals by the relative magnitudes of temporally-patterned responses. This may make it possible to estimate signal persistence and loop gains by observing decay rates of temporally-patterned neural responses following stimulus presentations. In NCC masking experiments that examine trials where targets are experienced or not, temporally-structured stimuli and their related neural signals. Studies of attentional mechanisms would benefit from being able to quantify the strengths of attribute-related signals in different attentional contexts. Studies of the contents of working memory and hippocampal replay might also similarly benefit if recall tasks and mazes used rhythmic cues rather than visual ones.

If the phase-locked or entrained rhythms can be observed in neural responses in different cortical regions, then it will be possible to determine if regeneration of specific neural signals is necessary for them to produce awareness of the patterns. Then one could test whether or to what degree particular brain regions must regenerate the pattern for it to be perceived. It would also be possible to determine when anesthetic agents suppress signals, disrupt inter-regional coordinations, and/or scramble them.

### 8.2 Generalizing to non-human systems

In investigating the neural basis of conscious awareness, we believe that we ought to begin with humans and animals, even including insects and cephalopods, because they show many publicly-observable signs of conscious awareness. These are anatomical, physiological, and behavioral third-person public observables that in humans correlate well with our first-person, privately-observable awarenesses. They are the means by which doctors assess awareness in humans who are incapable of verbal report, e.g., in minimally-conscious states and under different levels of anesthesia. We can cautiously infer that they have phenomenal awareness from those third-person correlates.

As members of a community of observers, we accept as empirical evidence, albeit with caution, the private, introspective observations and reports of each other’s individual awarenesses because we accept other humans as interchangeable members in that community of observers. Other humans are observers with whom we can reliably communicate through language, but humans are not the only possible observers, and communication of subjective states with some animals has been demonstrated, e.g., in parrots (Pepperberg, 1999, 2008).

We are not human exceptionalists, and we think even the ability to overtly report our subjective states is not required. Because signal regeneration is a more general, systems-theoretic hypothesis, other classes of organisms that have non-neural, regenerative signaling systems can also be considered. The hypothesis also suggests that artificial systems may also potentially produce awareness, but only if they are embedded (“embodied”) in a material substrate that is appropriately-organized (with closed loops and continuously regenerated signals), and, perhaps, not just digitally simulated.

As recurrent, regenerative signaling is likely a universal feature of virtually all nervous systems, if the hypothesis is sustained by introspecting human subjects, then it would suggest that all animals that have nervous systems with recurrent pathways that regenerate signals should also possess some degree of awareness. Insights can also be gained from the effects of anesthesia on animals across phyla (Mashour and Alkire, 2013). They and others (Roth, 2013) suggest that there is a phyletically-ancient neuroanatomical and neurocomputational Bauplan that encompasses almost all animals.

The vast majority of physical systems in the known universe do not appear to have organizational closure through self-production. They are not alive and do not appear to be sentient—the regenerative signal theory assumes that those that do must have evolved over time. This is compatible with emergentist views of consciousness but incompatible with panzoism (all matter alive) and most varieties of pan-psychism (all matter conscious).

## 9 Philosophical considerations

Some broader philosophical context is not required, but may be helpful. This section expresses the philosophical opinions of one of us (PC).

From a hylomorphic perspective, some system-properties stem from the *organization* of material processes (Modrak, 1987). These include physiological and informational functions on one hand and conscious awareness on the other. The perspective is not a Cartesian dualism of material and mental or spiritual substances, but a “one substance, multi-aspect” materialism. In this view there are as many aspects of material systems as there are alternative, complementary descriptions of them.

Awareness and its contents are held to be necessary concomitants of particular organizations of neuronal activity, i.e., no phenomenal experiences come without neuronal activity, and conversely, when particular organizations of activity are present, the corresponding particular experiences always come along with them.

Although there are pervasive correspondences between informational functions, such as short-term (e.g., echoic and working) memory, and awareness, along with IIT (Tononi et al., 2025) but in contrast with GNW and RP (Mudrik et al., 2025a), I take the position that awareness *per se* does not itself play a functional role. Both supervene on the organizations and actions of material systems. However, both short-term memory and awareness appear to be subserved by the same underlying neural mechanisms, such that they most often occur together (are highly correlated). However, for their characterizations, they require different sets of observables (Figure 1D) and different types of descriptions. In a number of circumstances, they can be separated (functions without awareness, awareness without function).

Many different material states discernable in public observables can produce each privately discernable phenomenal state (a many-to-fewer isomorphism relation), such that changes in these must be consequences of changes in material processes, but not necessarily the reverse. Contra dualist interactionism (Popper and Eccles, 1977) and some varieties of computational functionalism (Rose, 2006), the causal arrows run in only one direction.

I agree with Kim’s assertion that, in principle, the physical world is causally closed under physical laws (Kim, 1998), but this turns critically on one’s conception of “causation.” The term has multiple meanings (Ross and Bassett, 2024): Humean causality (regular temporal succession of events) vs. mechanistic causality (show us a concrete mechanism) vs. predictive correlations between observables (a la Granger causality). Arguably, mechanistic conceptions of causality cannot apply to relations between fundamentally different types of observables (e.g., public vs. private observables, physical vs. functional or physical vs. phenomenal observables, low-level and high-level observables in “top-down causation”), so mechanistic causation is not compatible with neuropsychological and neurophenomenological models until concrete, realistic neural mechanisms are incorporated to predict perceptual judgments and qualia matches. Causal mechanistic neurocomputational explanations will then become possible.

There are some parallels, between philosophies of consciousness and of life, that are worth mention. Questions of whether computer simulations of neural systems or regenerative self-production systems could have awareness are similar to ones debated in the early Artificial Life movement decades ago. Can life be created in a computer simulation? Ultimately the debates turned on the ontological assumptions of the different sides. Mostly mathematicians, mathematical physicists, and computer scientists held Platonic ontologies in which Forms are sufficient for everything, including life. They did not differentiate between computer simulations and material systems if, in some sense, they had the same essential form. On the other side, including myself, were theoretical biologists who held more Aristotelian, non-reductionist-materialist and hylomorphic ontologies in which specific types of Organization must be embedded in some material substrate (Organization + Matter) in order to produce systems that one would want to call living organisms. Many biologists tend to be reductionist materialists who believe that the particular molecular constituents of naturally-evolved organisms on Earth (nucleic acids, proteins, lipids, proteolipids, etc.) define what is life.

There is also a more general parallelism here with disagreements between ontologies of Platonic idealism, eliminative materialist ontologies, and Aristotelian hylomorphism. Computational functionalism (e.g., HOT theories) are Platonic theories based only on Form. Reductionist-materialist theories hold that biological neurons are essential for consciousness. Aristotelian hylomorphic frameworks, like my own, hold that appropriate organizations of signaling processes must be embodied in some kind of material substrate, whether neural or non-neural, to realize their functional and phenomenal properties.

## 10 Conclusion

Organization closure through active regeneration of sets of neural signals (“neural autopoiesis”) may provide a neural basis for conscious awareness. Proper structuring of neural signals may be critical for their regeneration, such that one mode by which general anesthetics could abolish awareness would be to scramble the neural signals. Scrambling could also disrupt signals that contain the contents of perception and internal control signals that coordinate neural processing. Along the lines of global neural workspace and recurrent processing theories, a time-domain based theory of regenerated temporally-coded signals in local and global circuits can be envisioned. These are new concepts in the neuroscience of consciousness.

Empirical testing of these ideas can be conducted. First correlations between neural responses and contents of awareness ought to be established. Then rhythm-tagged stimuli could be used to track neural signals through the system to determine the extents and durations of signal regenerations. Then manipulation of neural activity via anesthetics and electrical, magnetic, and optogenetic interventions can then be used to investigate causal relations and possible roles for neural codes. Resolving neural coding problems, understanding recurrent, regenerative processes, and further empirical explorations should help advance adequate, neurally-grounded theories of conscious awareness.
